# Agreement Between Spatiotemporal Gait Parameters Measured by a Markerless Motion Capture System and Two Reference Systems—a Treadmill-Based Photoelectric Cell and High-Speed Video Analyses: Comparative Study

**DOI:** 10.2196/19498

**Published:** 2020-10-23

**Authors:** Felipe García-Pinillos, Diego Jaén-Carrillo, Victor Soto Hermoso, Pedro Latorre Román, Pedro Delgado, Cristian Martinez, Antonio Carton, Luis Roche Seruendo

**Affiliations:** 1 University of Granada Granada Spain; 2 Universidad de La Frontera Temuco Chile; 3 Universidad San Jorge Zaragoza, Villanueva de Gallego Spain; 4 Universidad de Jaén Jaén Spain

**Keywords:** OptoGait, runners, sport technology, validity, gait, motion capture, video, feasibility, accuracy, Kinect

## Abstract

**Background:**

Markerless systems to capture body motion require no markers to be attached to the body, thereby improving clinical feasibility and testing time. However, the lack of markers might affect the accuracy of measurements.

**Objective:**

This study aimed to determine the absolute reliability and concurrent validity of the Kinect system with MotionMetrix software for spatiotemporal variables during running at a comfortable velocity, by comparing data between the combination system and two widely used systems—OptoGait and high-speed video analysis at 1000 Hz.

**Methods:**

In total, 25 runners followed a running protocol on a treadmill at a speed of 12 km/h. The Kinect+MotionMetrix combination measured spatiotemporal parameters during running (ie, contact time, flight time, step frequency, and step length), which were compared to those obtained from two reference systems.

**Results:**

Regardless of the system, flight time had the highest coefficients of variation (OptoGait: 16.4%; video analysis: 17.3%; Kinect+MotionMetrix: 23.2%). The rest of the coefficients of variation reported were lower than 8.1%. Correlation analysis showed very high correlations (*r*>0.8; *P*<.001) and almost perfect associations (intraclass correlation coefficient>0.81) between systems for all the spatiotemporal parameters except contact time, which had lower values. Bland-Altman plots revealed smaller systematic biases and random errors for step frequency and step length and larger systematic biases and random errors for temporal parameters with the Kinect+MotionMetrix system as compared to OptoGait (difference: contact time +3.0%, flight time −7.9%) and high-speed video analysis at 1000 Hz (difference: contact time +4.2%, flight time −11.3%). Accordingly, heteroscedasticity was found between systems for temporal parameters (*r*^2^>0.1).

**Conclusions:**

The results indicate that the Kinect+MotionMetrix combination slightly overestimates contact time and strongly underestimates flight time as compared to the OptoGait system and high-speed video analysis at 1000 Hz. However, it is a valid tool for measuring step frequency and step length when compared to reference systems. Future studies should determine the reliability of this system for determining temporal parameters.

## Introduction

The use of marker-based motion capture technology has increased significantly in both research and diagnostics. This is evident in its prominent use in the field of biomechanics. However, inherent limitations in data collection can preclude its employment in settings such as patient homes, sports fields, and other public areas where implementing an array of cameras proves problematic. A potential solution that has been suggested is the use of a markerless motion capture system [1,2].

Markerless systems do not require attaching any markers or sensors to the body, which substantially improves clinical feasibility and testing time. However, the lack of markers might affect the accuracy of measurements. Therefore, studies focused on analyzing the validity of these systems under different circumstances are especially important. In this context, a markerless motion capture system (ie, the Kinect) has received much attention from clinicians, sports practitioners, and researchers [1,3-9]. The Kinect sensor was originally designed for using body movement to interact with video games on the Microsoft Xbox platform. The system projects an infrared laser speckle pattern onto the viewing area of the infrared camera. This infrared camera detects the pattern and enables the creation of a 3D map by measuring deformations in the reference speckle pattern.

Previous studies have evaluated the validity of the Kinect sensor for the assessment of gait characteristics [1,3-7]. Among these studies, different pieces of software, including different filters and calibrators, have been examined. Clark et al [4] assessed the validity of the Kinect system with a customized software created in LabVIEW 2009 for examining the spatiotemporal characteristics of gait in 21 healthy individuals. In contrast, Lamine et al [7] compared the validity of the Kinect for gait kinematics analysis with that of the Vicon system, and a Cartesian calibration was performed for both motion capture devices. Similarly, Pfister et al [6] compared the concurrent validity of the Kinect with Brekel Kinect software for sagittal plane gait kinematics analysis with that of the Vicon Nexus. Dolatabadi et al [5] determined the concurrent validity of the Microsoft Kinect for Windows for measuring gait spatiotemporal parameters. Schmitz et al [1] tested the validity of the Kinect system with the KinectFusion software for kinematic data evaluation. All of these aforementioned studies included the use of the MotionMetrix software, which might have implications for the accuracy of measurements.

Since the validity of a gait analysis system is essential to determine whether the results are due to changes in gait pattern or simply systematic measurement errors, this study aimed to evaluate the absolute reliability and concurrent validity of the Kinect system with MotionMetrix software for measuring spatiotemporal variables during running at comfortable velocity (ie, 12 km/h) by comparing the data with two widely used systems—the OptoGait system and high-speed video analysis at 1000 Hz. Based on our previous experience with the Kinect+MotionMetrix combination system, we hypothesized spatiotemporal parameters to be similar to those reported by the OptoGait and high-speed video analysis systems.

## Methods

With the introduction of new systems, establishment of their reliability and validity is essential before practical use. In this study, the MotionMetrix system was compared to both high-speed video analysis system (1000 Hz) and the OptoGait system for measuring spatiotemporal parameters during a running protocol followed at a comfortable velocity.

### Participants

A group of 25 amateur endurance runners (male: n=22, 88%; female: n=3, 12%; age: mean 24 years, SD 6 years; height: mean 1.75 m, SD 0.07 m; body mass: mean 71 kg, SD 7.4 kg) voluntarily participated in this study. All participants met the following inclusion criteria: (1) age, ≥18 years, (2) ability to run 10 km in less than 50 min, and (3) absence of any injury (points 2 and 3 are valid for the 6 months before data collection). After receiving detailed information on the objectives and procedures of the study, each participant signed an informed consent form in order to participate, which complied with the ethical standards of the World Medical Association’s Declaration of Helsinki (2013). The participants were informed that they were free to leave the study at any time. The study was approved by the Ethics Committee of the University of La Frontera (Universidad de La Frontera, Temuco, Chile; Ref: 030_019).

### Procedures

Participants were individually tested on one specific day. Prior to all testing, participants refrained from vigorous physical activity for at least 48 hours, and all tests were performed at least 3 hours after a meal. Tests were performed with the participants’ usual training shoes to measure their typical performance.

Participants performed a running protocol on a motorized treadmill (Woodway Pro XL). The initial speed was set at 8 km/h, and the speed increased by 1 km/h each minute until a speed of 12 km/h was reached. Thereafter, in order to control the influence of running velocity on spatiotemporal parameters [10], the running velocity was fixed. Since previous studies [11,12] on human locomotion have shown that accommodation to running on a treadmill occurs at around 6 to 8 min, an 8-min accommodation program was performed at 12 km/h. Once the accommodation period was reached, recording started. The recording period lasted for 3 min and was performed at the same running velocity. Therefore, the entire running protocol lasted for 15 min. The slope was maintained at 0% over the entire protocol.

### Materials and Testing

#### Overview

Anthropometric data were measured using a precision stadiometer and balance (SECA 222 and 634). Spatiotemporal parameters measured during running included contact time (seconds), defined as time from when the foot contacts the ground to when the toes lift off the ground; flight time (seconds), defined as time from toe-off to initial ground contact of consecutive footfalls (eg, right-left); step length (meters), defined as length the treadmill belt moves from toe-off to initial ground contact in successive steps from forefoot to forefoot; and step frequency (steps per minute [SPM]), defined as number of ground contact events per minute. We used two different systems to measure these parameters—the OptoGait system versus high-speed video analysis at 1000 Hz. Both systems have the same temporal accuracy (±1 ms). Participants’ right legs were analyzed for temporal parameters in order to control potential influencing factors (ie, asymmetry) [13]. Further information about the systems are given below.

#### Microsoft Kinect

The Microsoft Kinect sensor (version 1.0, Microsoft) was designed to interact with video games through body movement. This sensor can track 3D motions via a depth sensor. It is also capable of locating 20 body joints in a 3D space at 30 Hz. We set two Microsoft Kinect sensors on either side of a treadmill in a specific configuration (170 cm from the center of the treadmill in the forward direction and 190 cm in the perpendicular direction from this point, according to the manufacturer guidelines). These sensors were used with MotionMetrix software (MotionMetrix AB). If both sensors were able to track the same point simultaneously (according to brand information), the Microsoft Kinect sensors could reach 60 Hz. Manufacturer recommendations were taken into account (ie, dynamic calibration provided by the software, tight clothes, no shiny black fabric or reflexes, no moving shoelaces, no moving hair, no sunlight, and no treadmill parts blocking the view of the runner). We recorded 30 s of data with this system (between minutes 1:30 and 2:00 of the recording period for each participant).

#### OptoGait

The OptoGait system detects any interruptions and therefore measures both contact time and flight time with a precision of 1/1000 seconds. Previous studies have analyzed the validity and reliability of this system during walking [14-18] and running [19]. Two parallel bars were placed on the lengthwise side edges of the treadmill at the same level as the contact surface, and the default filter setting of 0_0 (Gait R.in filter: 0 and Gait R.out filter: 0) was accepted. This setting indicates that contact time begins when more than 0 light-emitting diodes (LEDs) are activated (ie, when at least 1 LED is activated) and finishes when the number of LEDs activated return to 0. This setup has been shown to provide the smallest bias for temporal parameters in racewalking [20]. Spatiotemporal parameters (ie, contact time, flight time, step length, and step frequency) were measured for every step during the 30-second recording interval (between minutes 1:30 and 2:00 of the recording period for each participant).

#### High-Speed Video Analysis

High-speed video analysis has been shown to be a reliable and valid method for measuring running kinematics [21-24]. In this study, one experienced rater was involved in video analysis. In order to determine the test-retest reliability of the measurements, 10 recording intervals were analyzed on two different days, 24 hours apart, and an almost perfect agreement was found for all the spatiotemporal parameters measured (intraclass correlation coefficients [ICCs]>0.94). In this study, 2D video data were simultaneously collected at 1000 Hz using a high-speed camera (Imaging Source DFK 33UX174, The Imaging Source Europe GmbH). The range of interest was adjusted to achieve 1000 fps (784×144 resolution). The camera was placed perpendicular to the treadmill from a posterior view at 2 m from the center of the treadmill and at a height of 0.80 m. We recorded 30-s videos between minutes 1:30 and 2:00 of the recording period for each participant. Subsequently, videos were analyzed using the open license Kinovea software (version 0.8.27, Kinovea Open Source Project), and spatiotemporal parameters were determined. The contact time and flight time were calculated by identifying both the initial contact and take-off frames and counting the frames in between. Step length and step frequency were calculated as follows:

Step time (seconds) = flight time (seconds) + contact time (seconds) **(1)**

Step frequency (steps/second) = 1/step time (seconds) **(2)**

Step frequency (steps/minute) = 60 × step frequency (steps/second) **(3)**

Step length (meters) = running velocity (meter/minute)/step frequency (steps/min) **(4)**

### Statistical Analysis

Descriptive statistics are represented as mean and standard deviation. The normal distribution of the data and the homogeneity of variances were confirmed through the Shapiro-Wilk and Levene tests, respectively. Coefficients of variation (CVs, %) were calculated as a measure of absolute reliability [25,26]. To determine concurrent validity, a Pearson correlation analysis was performed between spatiotemporal parameters obtained from MotionMetrix and those obtained from the OptoGait system and video analysis. The following criteria were adopted to interpret the magnitude of correlations between measurement variables: <0.1 (trivial), 0.1-0.3 (small), 0.3-0.5 (moderate), 0.5-0.7 (large), 0.7-0.9 (very large), and 0.9-1.0 (almost perfect) [27]. The ICCs were also calculated between systems (MotionMetrix vs OptoGait and MotionMetrix vs video analysis) for spatiotemporal parameters during running. Based on the characteristics of this experimental design and the guidelines reported by Koo and Li [28], we decided to use a two-way random-effects model (ICC [2,k]), mean of measurements, and absolute definition for the ICC measurement. The interpretation of the ICC was based on the benchmarks reported by a previous study [29]: ICC<0 (poor), ICC 0-0.20 (slight), ICC 0.21-0.40 (fair), ICC 0.41-0.60 (moderate), ICC 0.61-0.80 (substantial), and ICC>0.81 (almost perfect). Additionally, the 95% CI of the ICC value was provided. Finally, Bland-Altman plots (ie, limits of agreement method; mean difference [1.96 SD]) [30] were constructed to examine the presence of systematic and proportional bias between video analysis at 1000 Hz and the OptoGait system and estimated values (ie, Kinect with MotionMetrix system) of spatiotemporal parameters during running. Heteroscedasticity of error was defined as *r*^2^>0.1 [25]. The level of significance used was *P*<.05. Data analysis was performed using SPSS (version 23).

## Results

Table 1 shows descriptive values of spatiotemporal parameters acquired from three different systems and the differences between systems (in absolute and relative values). As a measure of absolute reliability, Table 2 shows the CVs of spatiotemporal parameters obtained from the three different systems. The Kinect+MotionMetrix system obtained higher CVs than the reference systems for all the spatiotemporal parameters. Regardless of the system, flight time had the highest CVs (OptoGait: 16.4%; video analysis: 17.3%; Kinect+MotionMetrix*:* 23.2%). The rest of CVs reported were lower than 8.1%.

**Table 1:** Descriptive data of spatiotemporal parameters obtained from different systems (ie, Kinect with the MotionMetrix software, OptoGait, and high-speed video analysis at 1000 Hz).

Variable	OG^a^, mean (SD)	VA^b^, mean (SD)	MM^c^, mean (SD)	MM-OG difference, mean (%)^d^	MM-VA difference, mean (%)
Contact tine (s)	0.265 (0.015)	0.262 (0.013)	0.273 (0.021)	0.008 (3.0)	0.011 (4.2)
Flight time (s)	0.089 (0.018)	0.097 (0.017)	0.082 (0.019)	−0.007 (−7.9)	−0.011 (−11.3)
Step frequency (spm^e^)	166.60 (6.80)	165.76 (6.90)	167.23 (7.28)	0.63 (0.4)	1.47 (0.9)
Step length (cm)	115.53 (6.80)	116.58 (5.92)	115.44 (5.26)	−0.09 (−0.1)	−1.14 (−1.0)

^a^OG: OptoGait system.

^b^VA: High-speed video analysis.

^c^MM: Kinect system with the MotionMetrix software.

^d^%: Refers to values reported by the reference system.

^e^spm: Steps per minute.

**Table 2:** Coefficients of variation (%) of spatiotemporal parameters during running at 12 km/h obtained from three different systems.

Variable	OG^a^	VA^b^	MM^c^
Contact time, CV^d^	5.7	5.2	8.1
Flight time, CV	16.4	17.3	23.2
Step frequency, CV	4.1	4.2	4.4
Step length, CV	4.6	4.6	5.02

^a^OG: OptoGait system.

^b^VA: High-speed video analysis.

^c^MM: Kinect system with the MotionMetri*x* software.

^d^CV: Coefficient of variation.

A Pearson correlation analysis was conducted, and ICCs between systems, as well as their CIs, were calculated (Table 3). In the comparison between the OptoGait and Kinect+MotionMetrix systems, a significant substantial correlation (*r*>0.645; *P*<.001; ICC=0.712) was obtained for contact time, whereas significant, almost perfect correlations were found for flight time, step frequency, and step length (*r*>0.901; *P*<.001; ICCs>0.89). Similarly, the video analysis versus Kinect+MotionMetrix comparison showed a significant substantial correlation (*r*>0.664, *P*<.001; ICC=0.667) for contact time and significant, almost perfect correlations for the rest of variables (*r*>0.928; *P*<.001; ICCs>0.84).

**Table 3:** Pearson correlation analysis and intraclass correlation coefficients between spatiotemporal parameters obtained from the Kinect system with the MotionMetrix software and those obtained from two different systems (ie, OptoGait and high-speed video analysis) during running at a comfortable speed.

Variables	MM^a^ versus OG^b^			MM versus VA^c^		
	*r*	*P* value	ICC^d^ (95% CI)	*r*	*P* value	ICC (95% CI)
Contact time	0.645	<.001	0.712 (0.333-0.874)	0.664	<.001	0.667 (0.153-0.862)
Flight time	0.901	<.001	0.894 (0.420-0.967)	0.928	<.001	0.838 (0.159-0.961)
Step frequency	0.962	<.001	0.978 (0.951-0.990)	0.940	<.001	0.959 (0.873-0.984)
Step length	0.971	<.001	0.986 (0.968-0.994)	0.954	<.001	0.964 (0.881-0.986)

^a^MM: Kinect system with the MotionMetrix software.

^b^OG: OptoGait system.

^c^VA: High-speed video analysis.

^d^ICC: intraclass correlation coefficient.

**Figure 1:**
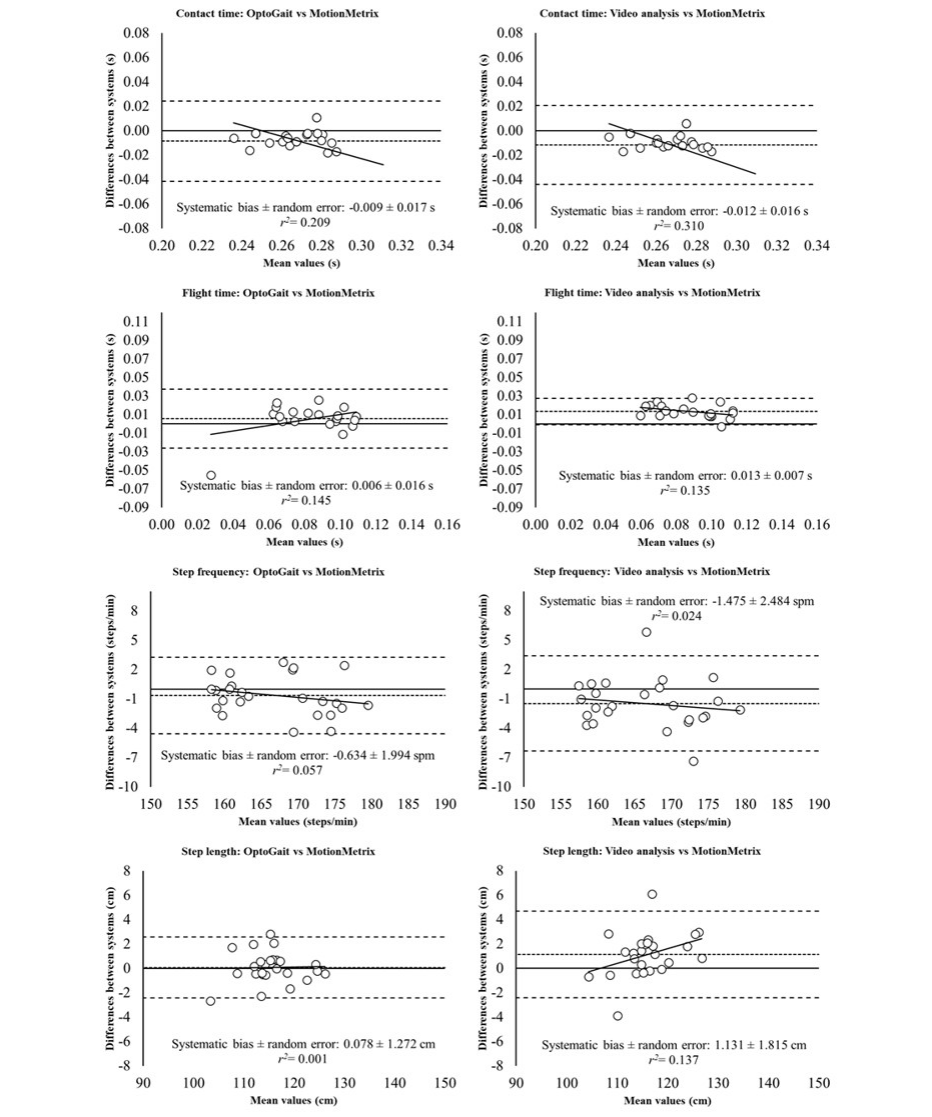
Bland-Altman plots for the measurement of spatiotemporal parameters (ie, from up to bottom: contact time, flight time, step frequency, and step length) during running at a comfortable speed obtained from Kinect with MotionMetrix software and two different systems (OptoGait and high-speed video analysis at 1000 Hz). The plot includes the mean difference (dotted line) and 95% limits of agreement (dashed line), along with the regression line (solid line).

Through Bland-Altman plots, the differences (systematic bias and random error) and the degree of agreement between systems (95% limits of agreement) were determined (Figure 1). The plots of OptoGait and Kinect+MotionMetrix revealed small systematic biases and random errors for step frequency and step length (step frequency difference: mean −0.634 steps per minute, SD 1.994 SPM; step length difference: mean 0.078 cm, SD 1.272 cm), but greater systematic biases and random errors for contact time and fight time (contact time difference: mean −0.009 s, SD 0.017 s; flight time difference: mean 0.006 s, SD 0.016 s) during running at a comfortable velocity. Accordingly, heteroscedasticity was found in temporal parameters (contact time and flight time, *r^2^*>0.1), whereas homoscedasticity was found in step frequency and step length (*r^2^*<0.1). Similarly, when data were compared between video analysis and the Kinect+MotionMetrix system, small systematic biases and random errors for step frequency and step length were found (step frequency difference: mean −1.475 SPM, SD 2.484 SPM; step length difference: mean 1.131 cm, SD 1.815 cm), whereas greater biases and errors were found for contact time and flight time (contact time difference: mean −0.012 s, SD 0.016 s; flight time difference: mean 0.013 s, SD 0.007 s). In this case, homoscedasticity was found only in step frequency (*r^2^*=0.024), whereas the rest of parameters revealed heteroscedasticity (*r^2^*>0.1).

## Discussion

This study aimed to evaluate the absolute reliability and concurrent validity of the Kinect with MotionMetrix software for measuring spatiotemporal variables during running at a comfortable velocity by comparing data from the combination system to data obtained from two widely used systems—the OptoGait system and high-speed video analysis at 1000 Hz. Our results showed that the Kinect+MotionMetrix combination reported higher CVs than the reference systems, even though values higher than 10% only were found for flight time, which is similar to the other systems tested in this study (ie, OptoGait and high-speed video analysis). Additionally, regarding the concurrent validity, an almost perfect level of agreement between systems was found for all the spatiotemporal parameters except contact time, which reported a moderate agreement.

There is limited research on the reliability and validity of markerless motion capture systems for measuring biomechanical parameters during walking or running on a treadmill. Some studies have examined the validity of the Kinect sensor for the assessment of gait characteristics [1,3-7], even though methodological inconsistencies (eg, software used, filters and calibrations applied, testing protocol used, and conditions present, etc) make comparisons difficult.

In that context, findings that have been reported about the validity of the Kinect system for measuring spatiotemporal parameters are controversial. A previous study [5] concluded that the Kinect for Windows is a valid tool for measuring the spatiotemporal parameters of gait during walking. Other studies [3,4] have reported important differences between spatiotemporal parameters measured from a 3D motion capture system and those measured from the Kinect system. Clark et al [4] indicated that the Kinect system reported lower values (ie, −16% step time, −19% stride time, and −1.7% step length) than a 3D system during walking. Similarly, Xu et al [3] indicated that the Kinect system reported valid step time and stride time values, but shorter stance times (ie, −9%) than a 3D system during walking. Therefore, it seems that the validity of the Kinect system for measuring spatiotemporal parameters is highly dependent on variables, such as the software and filter setting used, the gold standard or reference system tested, the protocol performed, and the target variables measured.

Since it has been demonstrated that running on a treadmill carries some biomechanical differences compared to above-ground running [31], caution must be taken when interpreting the results. Some of these studies, which were focused on determining the validity of the Kinect system, were conducted above the ground [4,5], while only 3 studies were conducted on a treadmill [3,6,7].

To the best of our knowledge, only one study [6] has examined the validity of the system during running. This is an important point because validity and reliability data during walking should not be extrapolated to running conditions, since the magnitude of the parameters change and different phases appear (ie, fight time does not exist during walking and there is no double-support time during running). The aforementioned study [6] examined sagittal plane gait kinematics with no mention of spatiotemporal parameters at different walking and jogging velocities (ie, walking velocity of 4.8 km/h increasing to a jogging velocity of 8.8 km/h) being lower than the velocity in this study (ie, 12 km/h), and the authors concluded that the measurement accuracy of the Kinect system was not acceptable for clinical measurement analysis (ie, the system did not provide consistent hip or knee measurements compared to a 3D system). Notably, Pfister et al [6] used old software combined with the Kinect system (ie, Brekel Kinect software), which might explain the differences between the Pfister et al [6] study and our study. The Brekel software worked at 30 Hz, while the software used in this study (ie, MotionMetrix) can reach 60 Hz. Therefore, a higher accuracy is expected with our study. Ours is the first study to examine the validity of the Kinect with MotionMetrix software for measuring spatiotemporal variables while participants ran on a treadmill at a comfortable velocity.

Ultimately, the main limitation of this study was the differences in the precision of the systems. Both the OptoGait system and high-speed video analysis captured data at 1000 Hz, whereas the Kinect with MotionMetrix software works at 60 Hz. This means that this system has a precision of 0.017 s, while the reference systems have a precision of 0.001 s. This point might explain the differences found in the CVs. Therefore, the differences between systems might be explained by the limitations of the markerless Kinect with MotionMetrix software.

In summary, our results indicate that the Kinect system with the MotionMetrix software slightly overestimates contact time and strongly underestimates flight time compared to both the OptoGait system (difference: contact time +3.0%, flight time: −7.9%) and high-speed video analysis at 1000 Hz (difference: contact time +4.2%, flight time: −11.3%). However, it is a valid tool for measuring step frequency and step length when compared to these reference systems (differences lower than 1%). Future studies should determine the reliability of this system for determining flight time with a CV of around 23%.

From a practical perspective, spatiotemporal gait characteristics are readily assessable by such software (ie, MotionMetrix) in conjunction with two Kinect sensors attached to a treadmill after a simple 30-second calibration, even though users must be aware of the characteristic of measurements. Further clinical implications of this system include low cost, time efficient, and wide availability.

## Abbreviations

CV: coefficient of variation
ICC: intraclass correlation coefficient
LED: light-emitting diode
SPM: steps per minute
